# P05.42. Student ranking of “evidence-informed practice guidelines” from NARCCIM workshop: “what are research literacy competencies for the CAM practitioner?”

**DOI:** 10.1186/1472-6882-12-S1-P402

**Published:** 2012-06-12

**Authors:** S Gomes

**Affiliations:** 1 Pacific College of Oriental Medicine, San Diego, USA

## Purpose

The purpose of this research was to have students rank the significance of the “Evidence-Informed Practice Guidelines” generated at the 2009 NARCCIM workshop to their actual practice.

## Methods

This was a qualitative survey ranking the 10 evidence-informed guidelines. This study was conducted from May 2009 to May 2011 and included 118 students.

## Results

Of the 10 evidence-informed guidelines list below, “Maintain ethical standards of practice” was rated the highest, with “Access relevant information to find evidence-informed answers to questions that arise in clinical practice” coming in second and “Integrate multiple forms of evidence into clinical practice” ranked third most significant.

1. Define EIP and describe its role

2. Describe fundamental principles of research

3. Generate searchable questions

4. Access relevant information to find evidence-informed answers to questions that arise in clinical practice.

5. Critically appraise different forms of evidence

6. Integrate multiple forms of evidence into clinical practice

7. Effectively integrates evidence into professional communications

8. Maintain ethical standards of practice

9. Engage in reflective practice

10. Participate in the culture of research

## Conclusion

Students preparing to graduate and start practice believe that it is most important to “Maintain ethical standards of practice”. The students frequently commented that this guideline does not fit well into what they believe is “evidence-informed practice” and it may need to be removed from the list. The need to “Access relevant information to find evidence-informed answers to questions that arise in clinical practice” was rated consistently high and should be used to inform the teaching and practice of research literacy.

